# Identification of D‐Fructose Dehydration Products by Infrared Multiphoton Dissociation Mass Spectrometry: The Spectral Signature of An Elusive 5‐Hydroxymethylfurfural Isomer

**DOI:** 10.1002/open.202500437

**Published:** 2025-09-25

**Authors:** Caterina Fraschetti, Massimiliano Aschi, Andreina Ricci, Roberta Astolfi, Antonello Filippi

**Affiliations:** ^1^ Dipartimento di Chimica e Tecnologie del Farmaco Sapienza Università di Roma Piazzale Aldo Moro 5 00185 Roma Italy; ^2^ Dipartimento di Scienze Fisiche e Chimiche Università degli Studi de L’Aquila Via Vetoio (Coppito 2) 67010 L’Aquila Italy; ^3^ Dipartimento di Matematica e Fisica Università della Campania L. Vanvitelli Viale Lincoln 5 81100 Caserta Italy

**Keywords:** 5‐hydroxymethylfuraldehyde, fructose dehydration, gas‐phase conformations, infrared multiphoton dissociation

## Abstract

A combined Electrospray Infrared MultiPhoton Dissociation Mass Spectrometry (ESI‐IRMPD‐MS) and Density Functional Theory (DFT) investigation has allowed to elucidate the structural features of the species arising from the triple dehydration of D‐fructose in the gas phase. The experimental workflow involves measuring and comparing the IRMPD spectra of different ionic populations: protonated 5‐hydroxymethylfurfural [**HMF**·H]^+^ and the ionic species coming from the triple dehydration of the ammonium‐D–fructose complex ([**Fru**·NH_4_]^+^). The IR‐photon induced fragmentation of [**Fru**·NH_4_]^+^ reveals the coexistence of two ionic populations, which arise from of two independent not intercrossing fragmentation pathways of the ionic precursor. One population exhibits an IRMPD spectrum matching with the ([**HMF**·H]^+^) one and corresponding to a carbonyl‐protonated structure. The second ionic product is its C2‐protonated protomer, which lies 75 kJ/mol above the global minimum. The presence of a less stable protomer is most likely due to its gas‐phase kinetic trapping. These findings contribute to a more refined understanding of gas‐phase carbohydrate dehydration and isomer formation at the molecular level.

## Graphical abstract

Two distinct ionic populations coexist during triple dehydration of ammonium‐D‐fructose, arising from independent, nonintercrossing fragmentation pathways: i) low energy population, carbonyl‐protonated structure; and ii) high energy population, C2‐protonated structure, which is kinetically trapped despite thermodynamic instability.

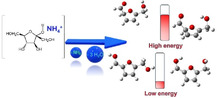

## 1 Introduction

**Scheme 1:**
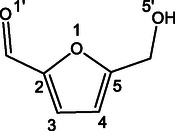
Structure of 5‐hydroxymethylfuraldehyde (5‐HMF).

5‐hydroxymethylfuraldehyde (5‐HMF, **Scheme** **1**) is a significantly small and relatively rigid organic molecule involved in a number of ongoing debates crossing different domains, i.e., food, pharmaceuticals, and biofuels fields. 5‐HMF can be considered a food quality marker, in particular for honey,^[^ ^1^, ^2^ ^–^ ^3^ ^]^ juice,^[^ ^4^, ^5^ ^–^ ^6^ ^]^ milk,^[^ ^7^, ^8^ ^–^ ^9^ ^]^ and coffee^[^ ^10^, ^11^ ^–^ ^12^ ^]^ matrices. The 5‐HMF impact on human health is known as highly toxic, due to its prompt absorption in the gastrointestinal tract, and its further metabolization to different derivatives exhibiting irritating and even mutagenic effects on the mucous membranes.^[^ ^13^ ^,^ ^14^ ^]^ It is well‐known that 5‐HMF is formed during thermal food processing through either the Maillard reaction or the acid‐catalyzed degradation of sugars, and the yield of its formation depends on the temperature and the medium acidity.^[^ ^15^ ^]^ 5‐HMF holds great relevance in pharmaceutics, due to its role as an intermediate in fine chemistry to achieve valuable chemicals in good yields and a sustainable way.^[^ ^16^, ^17^, ^18^, ^19^ ^–^ ^20^ ^]^ The sustainability is, in turn, the keyword to disentangle the information delivered by the great number of publications focusing on the chemistry of 5‐HMF. The production of carbon‐containing compounds from nonfossil sources has been for years a remarkable focus of researchers devoting great efforts in finding out and designing new technologies capable to employ renewable resources and scaling them up to an industrial perspective. Indeed, cellulose, a totally renewable feedstock, can be primarily hydrolyzed to cellobiose, and finally to glucose, which in turn isomerizes to fructose, the best candidate as 5‐HMF precursor.^[^ ^21^ ^,^ ^22^ ^]^ The mechanism of sugar degradation to 5‐HMF has been widely explored in condensed phase,^[^ ^23^, ^24^, ^25^ ^–^ ^26^ ^]^ but in the last decade it also stimulated gas phase studies performed by mass spectrometric (MS) approaches,^[^ ^27^ ^,^ ^28^ ^]^ which focus on the naked system at its molecular level. A MS investigation provides several important advantages enabling access to the intrinsic properties of naked species, and in parallel, allowing the experimental results to be compared with in vacuum calculations, which can provide very detailed information about the structural features of a molecular system. The MS study performed by Pepi et al.^[^ ^27^ ^]^ pointed out that the acid‐catalyzed gas phase dehydration of D‐fructose generates a species, which can be a protomer or an isomer of protonated 5‐HMF (herein indicated as [**HMF**·H]^+^). In a subsequent paper,^[^ ^28^ ^]^ Troiani et al. observed the presence of two fragmentation pathways in the ammonium‐catalyzed dehydration of D‐fructose, yielding two isobaric ionic products, i.e., the [**HMF**·H]^+^ and its isomer derivative, herein indicated as [**126**·H]^+^. The chemical identity of the [**126**·H]^+^ ion is still an experimentally elusive information due to the low MS structural resolving power. In this work, an Electrospray Infrared MultiPhoton Dissociation Mass Spectrometry (ESI‐IRMPD‐MS) approach is proposed to elucidate this challenging identification, taking advantage of a sensitive technique which is capable of elucidating very fine intra‐ or intermolecular features.^[^ ^29^ ^–^ ^31^ ^]^ The infrared multiphoton dissociation (IRMPD) spectrum of [**HMF**·H]^+^, arising from a 5‐HMF standard solution, has been compared with the spectrum obtained by isolating the [**126**·H]^+^ ion arising from the ammonium‐catalyzed dehydration of D‐fructose. The experimental spectra were finally validated through a theoretical investigation at the B3LYP‐D3/6‐311++G** level of theory.

## 2 Results and Discussion

### 2.1 Material and Methods

#### 2.1.1 IRMPD Experiments

5‐HMF was purchased from a commercial source and dissolved in methanol to 10^−4^ M final concentration without any further purification. The ESI‐generated ion cloud was introduced in a modified Bruker Esquire 6000 quadrupole ion trap and isolated using the standard Bruker Esquire Control (v6.2) software. ESI conditions used were as follows: syringe pump rate: 100 mLh^−^^1^; spray voltage: 3500 V; and capillary temperature: 250 °C. During the MS^2^ step, the mass‐selected ion cloud was irradiated, zeroing the excitation amplitude to avoid any collision‐induced dissociation (CID) process. Mass spectra were recorded in the standard mass range (*m/z* 50–3000 u) and normal scan‐mode resolution (13000 u s^−1^), with the accumulation time fixed at 0.5 s. The 2800–3700 cm^−1^ wavenumber range was explored using an Infrared (IR) OPO/OPA system of LaserVision, pumped by a 10 Hz Nd:YAG laser (Excel Technology Europe GmbH Surelite‐II, 650 mJ per pulse, 8 ns pulse duration). The output energy, measured between 3400 and 3600 cm^−1^, is about 20 mJ per pulse with a spectral bandwidth of ≈5 cm^−1^. The photon energy was increased at a rate of 0.1 cm^−1^ s^−1^. In the second experiment, commercial D‐fructose was dissolved in methanol to the final concentration of 10^−4^ M with the addition of 0.1% CH_3_CO_2_NH_4_. The [**Fru**·NH_4_]^+^ (*m/z* 198) was isolated and collisionally activated to induce its dissociation to the final [**126**·H]^+^ ion that was submitted to the multiphoton dissociation. The fragmentation efficiency was reported as the arbitrary unit R, defined as *R *= −log[(I_prec_)/(I_prec_+I_frag_)], in which I_prec_ is the intensity of the precursor ion and I_frag_ is the intensity of its fragment ion(s).

### 2.2 Computational Details

All the calculations were carried out with the Gaussian16 software 1 in the framework of density functional theory using the B3LYP hybrid functional 2 with empirical dispersion (B3LYP‐D3) in conjunction with the 6‐311++G** basis set. All the species were first optimized, then characterized as true minima by calculating the harmonic frequencies. The same frequencies were then utilized for modeling the spectra reported in the main text. For this purpose, all the peaks of interest were fitted to Gaussian functions with a sigma adapted for reproducing the experimental widths. Note that the spectral intensities, evaluated on the basis of the corresponding transition dipole moments, do not necessarily coincide with the experimental intensities. The modeled spectra should then be considered as only informative as far as the absorption frequencies are concerned. A value of 0.953 was used to scale the calculated frequencies based on the matching between the experimental and the calculated spectra. In particular, the fitting was based on the correspondence between the experimental band at 3662 cm^−1^, which cannot be other than the stretching of an unperturbed CH_2_ **O‐H**, and the most blueshifted calculated frequency of the four most stable calculated structures.

### 2.3 Results

#### 2.3.1 Electrospray Infrared MultiPhoton Dissociation Mass Spectrometry (ESI‐IRMPD) Spectra

**Figure 1:**
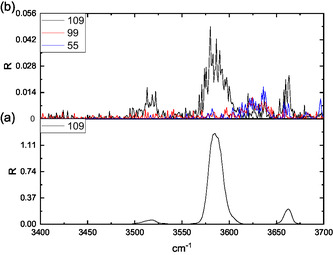
ESI‐IRMPD spectrum in the 3400–3700 cm^−1^ range of a) [**HMF**·H]^+^ ion (*m/z* 127) and b) [**126**·H]^+^ ion arising from the triple dehydration of D‐fructose**.**

**Figure 2:**
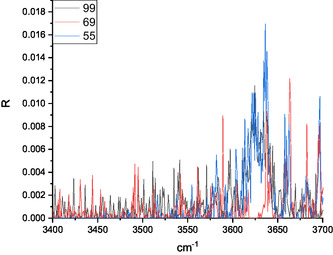
Ionic profile of the *m/z* 99, 69, and 55 products arising from the [**126**·H]^+^ ion IRMPD experiment (the further product having *m/z* 109 is not reported).

The ESI full spectrum of the 5‐HMF solution is dominated by the protonated [**HMF**·H]^+^ ion (*m/z* 127), followed by the less abundant [**HMF**·Na]^+^ sodiated species. The [**HMF**·H]^+^ ion generates the dehydrated derivative (*m/z* 109) as the only product ion, after the activation by either the collision with a buffer gas or by IR absorption in a multiphoton event (CID and IRMPD, respectively). Differently, the full spectrum resulting from electrospraying the methanol solution containing D‐fructose and 0.1% CH_3_CO_2_NH_4_ showed the intense peak of the [**Fru**·NH_4_]^+^ (*m/z* 198) ammonium complex, but not a detectable amount of the relevant [**Fru**·H]^+^ protonated ion (*m/z* 181), which is an unstable ionic species promptly undergoing to a first dehydration.^[^ ^27^ ^]^ Unfortunately, we could not reproduce the experimental workflow described in reference,^[^ ^28^ ^]^ since the intensity of the triply dehydrated fructose ion at *m/z* 127 isolated downstream of different MS^n^ pathways was too low to enable the acquisition of its IRMPD spectrum. We therefore directly isolated the [**126**·H]^+^ ion as the MS^2^ product of the [**Fru**·NH_4_]^+^ ion (*m/z* 198), which underwent a triple dehydration accompanied by ammonia loss. Besides the dehydrated product (*m/z* 109), both the CID and IRMPD experiments performed on the [**126**·H]^+^ ion arising from the [**Fru**·NH_4_]^+^ induced fragmentation, generated several product ions (*m/z* 99, 69, and 55). The different dissociation pattern of the isobaric [**HMF**·H]^+^ and [**126**·H]^+^ ions points to the presence of at least two different ionic populations, including an isomer or a protomer of 5‐HMF, whose structure can be experimentally probed through an IRMPD measurement. The IRMPD spectrum of [**HMF**·H]^+^ (**Figure** **1**a) is characterized by three bands in the 3490–3680 cm^−1^ range (namely, 3518, 3585, and 3662 cm^−1^). On the other side, the [**126**·H]^+^ IRMPD spectrum arising from the ammonium‐D‐fructose fragmentation (Figure 1b) can be clearly ascribed to the presence of at least two distinct ionic populations: the dehydrated one yielding the *m/z* 109 ion (black line in Figure 1b), and the product ions having *m/z* 55, 69, and 99 (Figure 1b). Beside the *m/z* 69 ion that was formed in a very poor concentration, the relative intensity of the 99 and 55 ions is basically constant throughout the investigated spectral range (**Figure** **2**). This evidence safely suggests that at least two protonated species, that could be isomers or protomers, were isolated and spectroscopically activated: one is responsible for the *m/z* = 109 product ion, meanwhile the second yields the *m/z* 55, 69, and 99 ionic triad. Moreover, the black profile in Figure 1b and the [**HMF**·H^+^] spectrum (Figure 1a) are perfectly superimposable, unequivocally indicating that the same [**HMF**·H^+^] ionic population is formed after the triple dehydration of D‐fructose. Finally, the most blueshifted absorption at 3662 cm^−1^, common to all the observed fragmentation channels (Figure 1a,b), is likely due to the stretching mode of an uncoordinated alcoholic group, therefore not diagnostic of the actual ionic structure.

#### 2.3.2 Calculated structures

**Figure 3:**
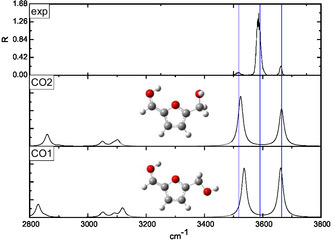
Comparison of the IRMPD spectrum of [**HMF**·H]^+^ ion with the calculated spectra of the **CO**1 and **CO**2 structures having *α *= 180° and *β *=  0°.

**Figure 4:**
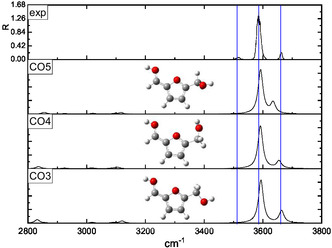
Comparison of the IRMPD spectrum of [**HMF**·H]^+^ ion with the calculated spectra of the **CO**3, **CO**4, and **CO**5 structures having *α *= 180° and *β *= 180°.

**Figure 5:**
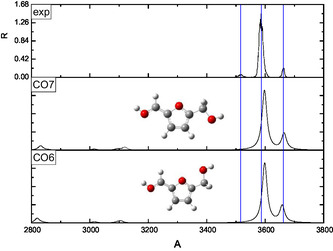
Comparison of the IRMPD spectrum of [**HMF**·H]^+^ ion with the calculated spectra of the **CO**6 and **CO**7 structures having *α *= 0° and *β *=  180°.

**Figure 6:**
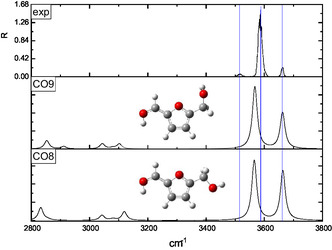
Comparison of the IRMPD spectrum of [**HMF**·H]^+^ ion with the calculated spectra of the **CO**8 and **CO**9 structures having *α *= 0° and *β *= 0°.

**Figure 7:**
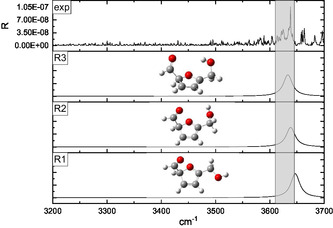
Comparison of the IRMPD spectrum of [**126**·H]^+^ ion with the calculated spectra of the **R**1, **R**2, and **R**3 C2‐protonated structures.

**Figure 8:**
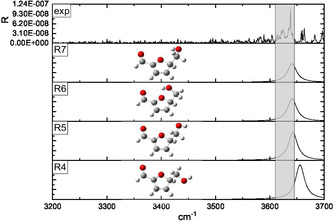
Comparison of the IRMPD spectrum of [**126**·H]^+^ ion with the calculated spectra of the **R**4, **R**5, **R**6, and **R**7 C5‐protonated structures.

**Figure 9:**
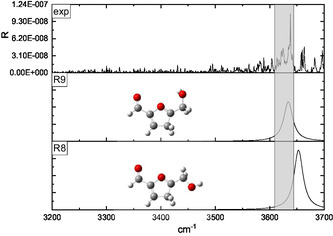
Comparison of the IRMPD spectrum of [**126**·H]^+^ ion with the calculated spectra of the **R**8 and **R**9 C4‐protonated structures.

**Figure 10:**
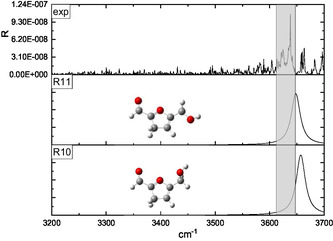
Comparison of the IRMPD spectrum of [**126**·H]^+^ ion with the calculated spectra of the **R**10 and **R**11 C3‐protonated structures.

The interpretation of the IRMPD spectra was supported by a conformational analysis performed at the B3LYP‐D3/6‐311++G** level of theory. Two families of protomers were investigated, namely carbonyl‐ and ring‐protonated structures **(CO**1–9 and **R**1–11, respectively, in **Figures** **3** **–** **10**) with the relevant energies reported in **Table** **1**. Concerning the **CO** *n* population (*n* = 1–9), the freedom degrees of the protonated carbonyl were explored by rotating the O1’‐C1’‐C2‐C3 (*α*) and H^+^‐O1’‐C1’‐C2 (*β*) dihedral angles that were let to assume both the 0° and 180° values (Figures 3, 4, 5–6 ). Moreover, the O5’‐C5’‐C5‐O1 (*γ*) dihedral angle, describing the position of the –CH_2_OH moiety with respect to the ring, was allowed to explore several values through different input structures (Figures 3–6). In the most stable structure **(CO**1), the *α–* *β–* *γ* triad responds to the 180°–0°–180° values pointing to a planar species that could involve an intramolecular folding favoring a weak proton‐O1 interaction (2.41 Å). Nevertheless, the DFT energy is not significantly affected by the 180° rotation of the H^+^‐O1’‐C1‐C2 angle **(CO**1 → **CO**3), pointing to a very weak intramolecular hydrogen bond interaction. The removal of the O5’···H4 interaction, accompanied by the O1/O5’ lone pairs facing destabilizes by several kJ/mol the ion **(CO**1 → **CO**2, **CO**3 → **CO**4, **CO7** → **CO**6, and **CO**8 → **CO**9 transitions); similarly, the **CO**3 → **CO**5 conversion, favoring the O5’/ring *π* system electrostatic repulsion, destabilizes by 16.2 kJ mol^−1^ the calculated structure. Finally, the 11.7 kJ mol^−1^ destabilization accompanying the **CO**7 → **CO**8 transition is most likely due to the H^+^···H3 strongly repulsive interaction. Concerning the ring‐protonated population (Figures 7, 8, 9–10 ), the most stable structure **(R**1) is a C2 protonated species lying 63.8 kJ mol^−1^ higher than **CO**1. The next C‐protonated structures destabilized by not less than 27.8 kJ mol^−1^ with respect to the **R**1 protomer.

**Table 1:** Calculated free energy of carbonyl‐ and ring‐protonated 5‐HMF optimized structures at B3LYP‐D3‐6‐311++G** level of theory.

Carbonyl‐protonated		Ring‐protonated	
Name	ΔG [kJ mol^−1^]	Name	ΔG [kJ mol^−1^]
**CO**1	0.0	**R**1	63.8
**CO**2	10.5	**R**2	75.1
**CO**3	0.6	**R**3	77.2
**CO**4	12.4	**R**4	107.8
**CO**5	16.8	**R**5	98.1
**CO**6	8.0	**R**6	91.6
**CO**7	1.4	**R**7	103.8
**CO**8	13.1	**R**8	93.8
**CO**9	24	**R**9	111.4
	–	**R**10	138.1
	–	**R**11	141.6

## 3 Discussion

### 3.1 IRMPD of [**HMF**·H]^+^ ion

Due to the remarkably higher basicity of the carbonyl oxygen, the IRMPD spectrum of the [**HMF**·H]^+^ ion was compared with the calculated spectrum of all the carbonyl protonated structures that in Figures 3–, 6 were clustered, according to specific conformational features of each species.

The spectra of the **CO**1–9 structures are basically dominated by the presence of two peaks, which are the spectral signatures of O1’–H^+^ and O5’–H stretching vibrational modes, the latter invariably blueshifted. The peaks below 3200 cm^−1^, having a definitely lower intensity, are due to the C–Hs stretching vibrations and were not detected in the experiment. The inspection of Figure 3–, 6 supports the hypothesis that the experimental band at 3515 cm^−1^ can be ascribed to the O1’–H^+^ stretching frequency of the **CO**2 conformer (3524 cm^−1^). Interestingly, the most stable conformer **CO**1, lying 10.5 kJ mol^−1^ below the **CO**2 one, appears not spectroscopically populated. The characteristics of the band at 3515 cm^−1^, which is low, large, and noisy, agree very well with the occurrence of a partial IRMPD ”transparency“^[^ ^32^ ^]^ due to the weak intramolecular interaction established between the proton and the O1 lone pairs. In the calculated spectra of the other carbonyl‐protonated structures **(CO**3–9 Figures 4–, 6 ), wherein the proton‐O1 interaction was removed, the O1’–H^+^ stretching is significantly blueshifted in a spectral region, which is experimentally dominated by the large band centered at 3584 cm^−1^. Indeed, the latter absorption can be easily assigned to the O1’–H^+^ stretching of the conformers included in the **CO**3–5 cluster (Figure 4), whose main structural feature is the *α *= 180° and *β *= 180° pair of values. The excellent superimposition of the calculated O5’–H stretching in the **CO**3 and **CO**4 conformers (3663 and 3654 cm^−1^, respectively) with the experimental most blue one (3660 cm^−1^) strongly suggests an important contribution to the gaseous population of the [**HMF**·H]^+^ ion arising from these structures, in particular, from the more stable **CO**3, which occupies a position very close to the global minimum (+0.6 kJ mol^−1^). Finally, based on a combination of spectroscopic and energy considerations, the carbonyl‐protonated species which populate the selected ion cloud were **CO**2 and **CO**3, which provided a very specific IR‐signature (Figures 3,4).

### 3.2 IRMPD of [**126**·H]^+^ ion

The IRMPD spectrum of the [**126**·H]^+^ ion, as resulting from the contribution of the *m/z* 99, 69, and 55 ionic profiles (Figure 2), was compared with the calculated spectrum of the ring‐protonated structures in Figures 7–, 10, wherein the protomers were clustered according to the protonation site. The 3200–3700 cm^−1^ region of all the calculated spectra is dominated by one band corresponding to the O5’–H stretching.

The comparison reported in Figures 7–, 10 points out to a very good superimposition of the noisy experimental band lying between 3620 and 3645 cm^−1^ and the absorptions of the **R**2 (3638 cm^−1^) and **R**3 (3644 cm^−1^) C2‐protonated structures and the **R**9 (3622 cm^−1^) C4‐protonated protomer. On one hand, we can rule out the presence of the **R**9 structure, due to its remarkable energy content (47.6 kJ mol^−1^ above the most stable ring‐protonated species), but it is worth noting that the most stable ring‐protonated structure **R**1, which is lower in energy by 11.3 kJ mol^−1^ than **R**2 one, does not seem spectroscopically relevant, due to its absorption which is blueshifted to 3658 cm^−1^.

**Scheme 2:**
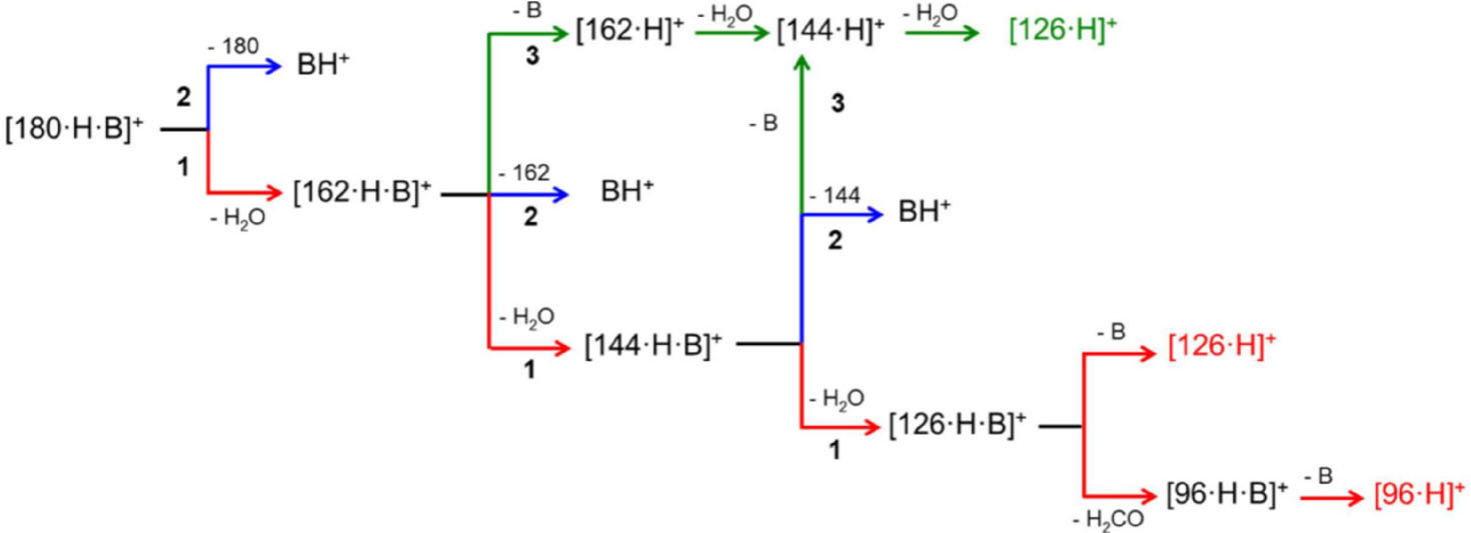
Fragmentation pathways of [**Fru**·NH_4_]^+^ adduct. In our study B = NH_3_ and [180·H·B]^+^ = [**Fru**·NH_4_]^+^. Reprinted with permission of A. Ricci (ChemistryOpen 2019, 8, 1190–1198).

Summarizing the spectroscopic evidences, the [**HMF**·H]^+^ ion isolated from a methanol 5‐HMF solution exists as a mixture of two carbonyl‐protonated structures, corresponding to the **CO**2 and **CO**3 conformers. These structures are representative of two conformational families differing in the *β* dihedral angle value. The presence of the **CO**4 structure within the gaseous population cannot be excluded if the strong spectral analogies between the **CO**3 and **CO**4 species are taken into account. On the other hand, as established by Troiani et al.^[^ ^28^ ^]^ the triple dehydration of fructose can be assisted by the ammonium ion exclusively in the first step (path 3 in **Scheme** **2**) or throughout the process (path 1 in Scheme 2). This branching, occurring at the [162·H·NH_3_]^+^ ion level, determines the formation of two final protomeric populations, which exhibit a different MS2 fragmentation pattern. Our ESI‐IRMPD workflow collected both the ionic populations downstream the 1/3 pathways and discriminated them based on the different spectroscopic signatures, i.e., the carbonyl‐protonated **CO**2/**CO**3 conformers (final product of path 1 in Scheme 2) and the C2‐protonated **R**2/**R**3 species (final product of path 3 in Scheme 2). It is worth noting that in both protomer populations, we did not observe the signature of the most stable structures, which are **CO**1 and **R**1 protomers, 10.5 and 11.3 kJ mol^−1^ lower in energy than the detected conformers, respectively. The absence of the **CO**1 conformer in the measured IRMPD spectrum could be ascribed to the preferential formation of other isomers, slightly less stable, which cannot interconvert to the more stable gaseous counterpart after the release in the gas phase.^[^ ^30^ ^,^ ^33^, ^34^, ^35^ ^–^ ^36^ ^]^ The formation of these species could result from a kinetic trapping assisted by the solvent during the electrospray of the 5‐HMF solution and/or by NH_3_ during the gas‐phase dehydration of the [**Fru**·NH_4_]^+^ ion.^[^ ^37^ ^]^ Furthermore, the **R**2/**R**3 ring‐protonated species arise from the triple fructose dehydration pathway assisted by the ammonium ion in the gas phase,^[^ ^28^ ^]^ which occurs along a different reaction coordinate to a final ionic population not interconverting to the most stable **R**1 conformer. In both cases, a rotation around the *γ* angle triggering the **CO**1 ⇄ **CO**2 and **R**1 ⇄ **R**2 conversions, most likely involves a 20–25 kJ mol^−1^ torsional barrier,^[^ ^38^ ^]^ which cannot be considered a completely free rotation in our experimental conditions. A further hypothesis, hard to experimentally validate, could be the involvement of a remarkable difference in the dipole moment variation during the vibrational excitation, which can enable the absorption of one specific rotamer, even if it is not the lower energy species.^[^ ^37^ ^]^

## 4 Conclusion

The products of the triple dehydration of ammonium‐D‐fructose have been probed through ESI‐IRMPD‐MS measurements. Primarily, the measurement of the IRMPD spectrum of protonated 5‐HMF indicated the coexistence of two carbonyl‐protonated conformers, both characterized by *α *= 180°, but differing by the value of the *β* angle, which can be 0° or 180° in **CO**2 and **CO**3, respectively. The ionic species isolated downstream of the triple dehydration pathway of the ammonium‐D‐fructose adduct consist of two isomeric ionic populations: the protonated 5‐HMF **(CO**2 and **CO**3 rotamers) and a C2‐ring protonated one **(R**2 and **R**3 species), which lies very high in energy if compared to the carbonyl‐protonated structures (+75.1 kJ mol^−1^ above the global minimum). The formation of a structure with a very high energy content is most likely due to its kinetic trapping along a reaction coordinate, which does not allow the ionic population to evolve to more stable species, surmounting a definitely high activation barrier (about 125.5 kJ mol^−^^1^).^[^ ^39^ ^]^ The coexistence of two protomeric populations agrees with the occurrence of two different fragmentation pathways differing in the sequence of neutral loss and then in the specific role of the ammonium cation.^[^ ^28^ ^]^

## Supporting Information

Data supporting the discussion within this study are available in the supplementary material of this article.

## Conflict of Interest

The authors declare no conflict of interest.

## Author Contributions

**Caterina Fraschetti**: conceptualization (lead); investigation (lead); writing—original draft (lead); writing—review & editing (lead). **Massimiliano Aschi**: software (lead). **Andreina Ricci**: conceptualization (supporting): data curation (supporting). **Antonello Filippi**: conceptualization (supporting); investigation (supporting); methodology (supporting).

## Supporting information

Supplementary Material

## Data Availability

The data that support the findings of this study are available from the corresponding author upon reasonable request.
