# Supplementary material for: Identification of D‐Fructose Dehydration Products by Infrared Multiphoton Dissociation Mass Spectrometry: The Spectral Signature of An Elusive 5‐Hydroxymethylfurfural Isomer
Source: ChemistryOpen. 2025 Sep 25;14(12):e202500437. doi: 10.1002/open.202500437 (PMC12680562; doi:10.1002/open.202500437)
Supplement: Supplementary file 1 — Supplementary Material [file OPEN-14-e202500437-s001.pdf]

Identification of D-Fructose Dehydration Products by Infrared Multi-Photon Dissociation Mass Spectrometry: The Spectral Signature of an Elusive 5-Hydroxymethylfurfural Isomer

Caterina Fraschetti,* Massimiliano Aschi, Andreina Ricci, Roberta Astolfi, and Antonello Filippi

Optimized Cartesian coordinates in XYZ format for all molecular structures reported in this work. All geometries were optimized at the B3LYP-D3/6-311++G(d,p) level of theory

CO1

16

O	1.297162	-0.000169	1.777208
C	0.017536	-0.000126	1.240907
C	0.114708	-0.000050	-0.160444
C	1.468741	0.000059	-0.470964
C	2.147689	-0.000057	0.744488
C	-1.062409	-0.000006	2.087679
O	-1.018651	0.000095	3.387960
C	3.602668	-0.000127	1.039280
O	4.261652	0.000343	-0.202067
H	-2.068887	0.000035	1.682164
H	-0.719858	-0.000036	-0.846676
H	1.941622	0.000195	-1.439349
H	3.828068	0.886217	1.653021
H	3.828079	-0.886963	1.652313
H	5.216127	0.000502	-0.073273
H	-0.113306	0.000087	3.743693

CO2

16

C	1.025487	0.469973	0.019144
O	-0.014289	-0.360804	0.024514
C	-1.160819	0.411725	-0.017977

C	-0.791812	1.766898	-0.050575
C	0.595656	1.800116	-0.025796
C	-2.379485	-0.222439	-0.010793
O	-2.567416	-1.505949	0.035845
C	2.400426	-0.119451	0.124531
O	2.409709	-1.511797	-0.031988
H	-3.296620	0.356741	-0.043413
H	-1.471168	2.606138	-0.090586
H	1.233021	2.670540	-0.041991
H	2.765118	0.110811	1.135569
H	3.063715	0.410511	-0.568740
H	2.684316	-1.751050	-0.922751
H	-1.739121	-2.016220	0.073728

CO3

16

O	1.288870	0.000081	1.776079
C	0.013269	0.000043	1.242627
C	0.110579	0.000106	-0.164136
C	1.460990	0.000016	-0.473870
C	2.135674	0.000059	0.748925
C	-1.084879	-0.000025	2.059056
O	-0.967786	-0.000050	3.359584
C	3.588844	0.000020	1.048643
O	4.256656	0.000388	-0.188843
H	-2.073055	-0.000028	1.605195
H	-0.723536	0.000117	-0.850856
H	1.936796	0.000007	-1.440660
H	3.809500	0.885738	1.664753
H	3.809506	-0.886047	1.664253
H	5.209858	-0.000376	-0.051978
H	-1.821681	-0.000052	3.817166

CO4

16

C	1.423148	0.071200	-0.396873
C	2.037413	0.000735	0.859978
O	1.131317	-0.039834	1.831210
C	-0.112861	0.009255	1.234123
C	0.058545	0.075353	-0.163722
C	3.482752	-0.050880	1.250019
O	3.729227	0.469961	2.526176
C	-1.252481	0.004423	1.992732
O	-1.203283	-0.053450	3.295214
H	-2.215267	0.048189	1.488866
H	-0.735949	0.130046	-0.893846
H	1.932665	0.123918	-1.346095
H	4.046094	0.543866	0.524400
H	3.821463	-1.092157	1.129004
H	3.589943	-0.199915	3.204101
H	-2.079068	-0.044440	3.709576

CO5

16

C	0.798903	1.451970	0.124552
C	0.968779	0.075570	0.276983
O	-0.196994	-0.564193	0.195689
C	-1.168524	0.390216	-0.012100
C	-0.558373	1.659124	-0.058525
C	2.207763	-0.737514	0.459994
O	2.985503	-0.529121	-0.707594
C	-2.483956	0.033522	-0.148106
O	-2.862765	-1.211285	-0.069367
H	-3.219857	0.813376	-0.327220
H	-1.069878	2.597759	-0.210223
H	1.588931	2.184644	0.141521
H	1.931909	-1.787090	0.596626
H	2.724435	-0.382888	1.363012

H	3.868232	-0.898339	-0.582742
H	-3.817277	-1.327996	-0.187577

CO6

16

O	-2.264990	0.078597	1.707104
C	-1.027627	0.178474	2.121159
C	0.005920	0.095940	1.229564
C	0.096698	-0.088953	-0.164504
C	1.443196	-0.084403	-0.476204
C	2.130642	0.103276	0.734033
C	3.591133	0.193955	1.005230
O	3.797656	0.364100	2.379556
O	1.288530	0.210899	1.746558
H	-2.910196	0.151785	2.425522
H	-0.815706	0.327385	3.177926
H	-0.740704	-0.209270	-0.835344
H	1.896570	-0.200633	-1.448280
H	4.741860	0.415630	2.563274
H	3.979432	1.030184	0.400283
H	4.051843	-0.724074	0.605044

CO7

16

C	2.135797	0.007929	0.753566
O	1.293372	0.014383	1.780103
C	0.010200	0.054023	1.246587
C	0.106807	0.071691	-0.158747
C	1.456008	0.042374	-0.468219
C	-1.033266	0.067393	2.130444
O	-2.265550	0.104475	1.692119
C	3.591142	-0.032470	1.044524

O	4.249720	-0.032934	-0.197948
H	-0.836687	0.047211	3.200324
H	-0.731255	0.102354	-0.839195
H	1.930898	0.044272	-1.435680
H	3.838906	0.839896	1.669204
H	3.794448	-0.931934	1.646375
H	5.203666	-0.057634	-0.068761
H	-2.920938	0.110677	2.405077

CO8

16

C	2.149199	0.026159	0.745858
O	1.296957	0.051323	1.761481
C	0.018085	0.141958	1.222021
C	0.131996	0.171181	-0.181922
C	1.482147	0.097868	-0.481095
C	-1.014707	0.181579	2.125359
O	-2.288194	0.264602	1.863177
C	3.600027	-0.069020	1.047747
O	4.266002	-0.075741	-0.190783
H	-0.796883	0.141872	3.188536
H	-0.680863	0.238325	-0.891000
H	1.963161	0.093995	-1.445529
H	3.873300	0.785612	1.685990
H	3.766964	-0.983204	1.638391
H	5.217689	-0.137737	-0.056874
H	-2.506068	0.300394	0.918241

CO9

16

C	0.438074	1.678359	-0.038844
C	1.092125	0.441525	0.061609
O	0.214784	-0.548674	0.101998
C	-1.054883	0.006916	0.024570
C	-0.918328	1.406568	-0.060173

C	2.551622	0.101334	0.129530
O	2.824721	-1.206878	-0.286394
C	-2.099921	-0.885102	0.029491
O	-3.371880	-0.614887	-0.031630
H	-1.892260	-1.949181	0.090840
H	-1.716592	2.130826	-0.138863
H	0.916091	2.643828	-0.097434
H	2.901435	0.315870	1.149912
H	3.082420	0.795917	-0.530669
H	2.901119	-1.801103	0.467008
H	-3.585347	0.329761	-0.089684

R1

16

O	1.447268	0.333926	1.840408
C	0.080943	0.181244	1.411343
C	0.187721	-0.023222	-0.049710
C	1.501703	-0.116345	-0.379301
C	2.226443	0.093525	0.828740
C	-0.505567	-1.130157	2.096130
O	0.209391	-2.008606	2.446436
C	3.692024	0.073194	1.046532
O	4.293987	-0.109561	-0.207561
H	-1.605503	-1.132738	2.177611
H	-0.665790	-0.134539	-0.705866
H	1.959621	-0.306393	-1.337194
H	3.972723	1.016275	1.542120
H	3.898779	-0.739338	1.762381
H	5.249885	-0.187771	-0.114438
H	-0.487461	1.054358	1.741142

R2

16

O	1.358125	0.423253	1.804761
C	0.011784	0.188425	1.350343

C	0.168318	-0.087497	-0.094937
C	1.492609	-0.140124	-0.379603
C	2.179559	0.171289	0.835845
C	-0.529782	-1.108392	2.095144
O	0.218402	-1.932094	2.505936
C	3.647295	0.237202	1.097601
O	3.985828	0.779415	2.331699
H	-1.629613	-1.159199	2.158093
H	-0.658638	-0.266358	-0.769694
H	1.965247	-0.360726	-1.325100
H	4.086054	0.866355	0.311697
H	4.043328	-0.777634	0.918913
H	3.894546	0.129266	3.038296
H	-0.601106	1.052617	1.618264

R3

16

C	1.155512	0.865250	1.699869
C	0.093504	0.282586	1.090090
C	0.553938	-0.156599	-0.190339
O	1.797269	0.150585	-0.387701
C	2.334442	0.718967	0.821348
H	1.187802	1.306230	2.687542
H	-0.909913	0.158854	1.469269
C	-0.201042	-0.869181	-1.263315
H	-1.035100	-0.210093	-1.559688
H	-0.671832	-1.740263	-0.786569
O	0.583154	-1.319730	-2.318364
H	0.738161	-0.623204	-2.966405
C	3.334096	-0.358360	1.452704
H	4.144910	0.086338	2.053227
O	3.158349	-1.517306	1.284023
H	2.882414	1.628749	0.568144

R4

16

O	1.273861	0.561742	1.781328
C	0.173602	0.097941	1.286513
C	0.238168	-0.123532	-0.112745
C	1.500447	0.230666	-0.489323
C	2.241976	0.684250	0.701782
C	-0.992335	-0.142044	2.236078
O	-0.869975	0.054809	3.408393
C	3.502798	-0.158612	1.039140
O	4.276362	-0.091639	-0.133674
H	-1.916494	-0.501461	1.753789
H	-0.564900	-0.506155	-0.725282
H	1.946798	0.186683	-1.474035
H	3.987301	0.282481	1.914483
H	3.188820	-1.181645	1.276183
H	5.213505	-0.193126	0.066360
H	2.521774	1.742746	0.643225

R5

16

O	1.108602	0.348428	1.836253
C	0.028824	0.033571	1.205504
C	0.190852	0.030610	-0.206086
C	1.489330	0.376833	-0.431701
C	2.166931	0.567714	0.861052
C	-1.217336	-0.279387	2.021220
O	-1.190502	-0.234588	3.214889
C	3.339646	-0.439570	1.179209
O	3.828169	-0.235148	2.461607
H	-2.107641	-0.538152	1.423997
H	-0.579404	-0.199147	-0.927558
H	1.980906	0.482997	-1.390418
H	4.085578	-0.313594	0.387427
H	2.926964	-1.449268	1.124372
H	4.591582	0.353568	2.461489
H	2.531040	1.587551	1.024589

R6

16

C	2.176284	0.731190	0.657350
O	1.163554	0.787470	1.712936
C	0.089220	0.231202	1.273552
C	0.186511	-0.195616	-0.078414
C	1.448606	0.115256	-0.475130
H	1.899700	-0.071222	-1.441126
H	-0.597689	-0.677513	-0.643068
C	-1.095083	0.111615	2.224149
O	-1.013722	0.511112	3.347262
H	-1.991009	-0.363100	1.791031
H	2.485703	1.765743	0.469524
C	3.373716	-0.087345	1.178522
H	3.714679	0.369327	2.113128
H	4.168014	-0.003332	0.426402
O	2.912509	-1.407900	1.340557
H	3.503038	-1.904815	1.917562

R7

16

C	0.002399	0.107634	0.035755
C	-0.005548	0.007555	1.500874
C	1.280985	-0.029637	1.954574
C	2.100946	0.053339	0.800653
O	1.422018	0.153284	-0.294457
H	-0.441705	1.029242	-0.356346
C	-0.644288	-1.087802	-0.768109
H	-1.681313	-1.152185	-0.431435
O	-0.642131	-0.814178	-2.127399
H	-0.118609	-2.009139	-0.496402
H	-0.910361	-0.034226	2.094106
H	1.633077	-0.109936	2.972509
C	3.618310	0.025085	0.702065
H	4.147727	0.033811	1.669236
O	4.155579	-0.009748	-0.365346
H	0.178887	-1.100114	-2.544424

R8

16

O	1.353998	-0.020964	1.754374
C	0.017408	0.007076	1.205896
C	0.092645	0.063040	-0.125059
C	1.536153	0.074457	-0.514203
C	2.200442	0.016851	0.801553
C	-1.082229	-0.031471	2.202197
O	-0.872690	-0.082448	3.382744
C	3.650788	0.001645	1.086307
O	4.305843	0.039359	-0.152151
H	-2.091370	-0.009317	1.753672
H	-0.743351	0.094543	-0.807887
H	1.864489	-0.768154	-1.142162
H	3.867447	0.865578	1.737752
H	3.862888	-0.901966	1.682515
H	5.262595	0.038074	-0.033439
H	1.870469	0.964022	-1.070005

R9

16

O	1.221735	-0.070211	1.709393
C	-0.099695	-0.000607	1.129851
C	0.009244	0.067247	-0.196688
C	1.464374	0.038758	-0.553095
C	2.100685	-0.040396	0.788916
C	-1.224169	-0.010100	2.100146
O	-1.042048	-0.046473	3.285366
C	3.545405	-0.075827	1.155254
O	3.798874	-0.501161	2.450120
H	-2.221931	0.022866	1.627784
H	-0.807094	0.127634	-0.901304
H	1.748721	-0.834365	-1.159334
H	3.944381	0.928159	0.911183
H	4.047437	-0.761122	0.459973

H	3.633312	0.193719	3.098955
H	1.811737	0.914425	-1.118552

R10

16

O	1.249786	-0.031793	1.741149
C	0.055090	0.068830	1.302841
C	0.051508	0.224603	-0.160692
C	1.507116	0.193582	-0.484752
C	2.180171	0.041626	0.659437
C	-1.094453	0.016044	2.289977
O	-0.881127	-0.116444	3.459795
C	3.616249	-0.064957	1.035494
O	4.317806	0.013324	-0.183651
H	-2.096785	0.107939	1.840703
H	-0.541906	-0.575929	-0.633781
H	1.952609	0.276809	-1.463432
H	3.863677	0.751301	1.729654
H	3.775660	-1.015431	1.565264
H	5.267304	-0.018915	-0.023240
H	-0.463932	1.156131	-0.449497

R11

16

O	1.083993	0.261870	1.750157
C	-0.085217	0.203141	1.257711
C	-0.033648	0.023733	-0.204712
C	1.437238	-0.006348	-0.458004
C	2.072568	0.133785	0.707302
C	-1.283665	0.332359	2.184276
O	-1.124500	0.546744	3.349401
C	3.477013	0.118110	1.189864
O	3.616328	-1.062143	1.959136
H	-2.264504	0.218560	1.694262
H	-0.556136	-0.902036	-0.498895
H	1.903923	-0.138805	-1.422017

H	3.663734	1.026796	1.777495
H	4.132695	0.134272	0.310621
H	4.420858	-1.015913	2.488247
H	-0.580537	0.830262	-0.719213

R12

16

O	1.304654	-0.121824	1.653176
C	-0.041129	-0.058088	1.172137
C	0.064603	0.023412	-0.171635
C	1.459491	0.070762	-0.544657
C	2.212027	0.005678	0.568899
C	-1.033722	-0.053265	2.249717
O	-0.658337	-0.044826	3.400389
C	3.638498	0.009707	1.015964
O	3.576138	0.409946	2.383151
H	-2.090557	-0.047748	1.943431
H	-0.773229	0.060417	-0.851953
H	1.842693	0.152213	-1.550042
H	4.207908	0.710385	0.396450
H	4.068150	-0.993825	0.905009
H	4.424596	0.292184	2.825308
H	1.598382	0.090759	2.578331
